# Bullous Systemic Lupus Erythematosus: A Case Report

**DOI:** 10.7759/cureus.40002

**Published:** 2023-06-05

**Authors:** Ganesh Giri, Sepideh Bagheri

**Affiliations:** 1 Medicine, Charles E Schmidt College of Medicine, Florida Atlantic University, Boca Raton, USA; 2 Dermatologist, Kaiser Permenente, Sacramento, USA

**Keywords:** vesicles, skin, blisters, lupus, bullous lupus

## Abstract

Bullous systemic lupus erythematous (BSLE) is a very rare autoimmune disease characterized by vesiculobullous lesions on mostly sun-exposed areas of skin. We present a case of a 36-year-old female who developed vesiculobullous lesions after previously having poorly controlled lupus. Dapsone was added to her treatment plan, and the lesions healed in a few weeks without scarring or pigmentation.

## Introduction

Bullous systemic lupus erythematosus (BSLE) is a very rare dermatologic manifestation of systemic lupus erythematosus (SLE) due to autoantibodies to collagen VII in the dermo-epidermal junction of the skin [1]. The lesions appear acutely as tense clear fluid-filled vesicles over erythematous plaques in mostly sun-exposed skin on the face, neck, trunk, upper extremities, oral mucosa, and the vermillion border [2]. 

Mucous membrane lesions are seen in 50% of patients and renal involvement in 50% of patients. Histological characteristics of BSLE include subepidermal bullae with a nuclear dusting of neutrophils in the upper dermis, abundant mucin deposits, and a lack of eosinophils [3].

Direct immunofluorescence shows linear to granular deposits of IgG, IgA, and IgM immune complexes and complements along the basal membrane. Indirect immunofluorescence shows auto-antibodies to dermal-epidermal junction collagen VII or basal membrane zone or epidermis [1]. The lesions do not respond to oral steroids. Around 90% of patients respond to dapsone [4]. The lesions heal in a few weeks without scarring or milia, but pigment changes may occur [5]. 

Bullous systemic lupus erythematosus does not correlate with the severity of lupus but seems to be associated with active lupus nephritis [3]. It is seen in less than 1% of patients with lupus. It is mostly seen in patients with known lupus but sometimes may be the initial manifestation of lupus. Bullous systemic lupus erythematosus is seen in all races, but it is more common in women aged between 20 and 40 years and of African-American descent [2]. 

We report a case of BSLE in a 36-year-old patient with a history of poorly controlled lupus. Due to the presence of worsening lupus nephritis in addition to the skin lesions, the patient was initially treated with azathioprine, prednisone, and mycophenolate. However, the skin lesions did not respond. The patient responded well to dapsone as expected as it is the drug of choice for treating BSLE skin lesions [4]. 

## Case presentation

The patient is a 36-year-old female diagnosed and being treated for lupus for one year. At the initial diagnosis of lupus, she was started on hydroxychloroquine 400 mg and oral prednisone 60 mg taper. Her symptoms included fatigue, arthralgia, intermittent oral ulcers, Raynaud’s syndrome, biopsy-proven leukocytoclastic vasculitic rash over the lower extremities, and recurrent pleurisy. The rash resolved and other symptoms improved. As the prednisone dosage was decreased, her symptoms flared up and the prednisone dosage was titrated a few times. As her symptoms were recurrent and laboratory work showed elevated antibody markers, she was started on colchicine 0.6 mg twice a day, followed by azathioprine 100 mg.

**Figure 1:**
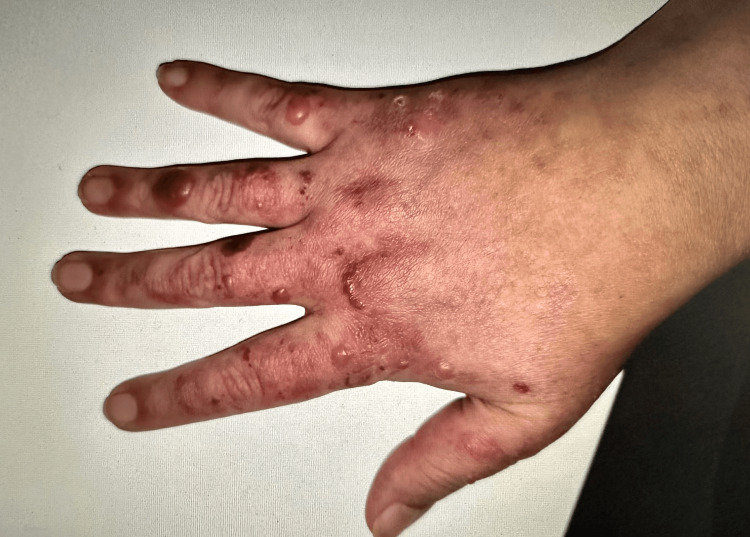
Vesiculobullous lesions on the dorsum of the hand

**Figure 2:**
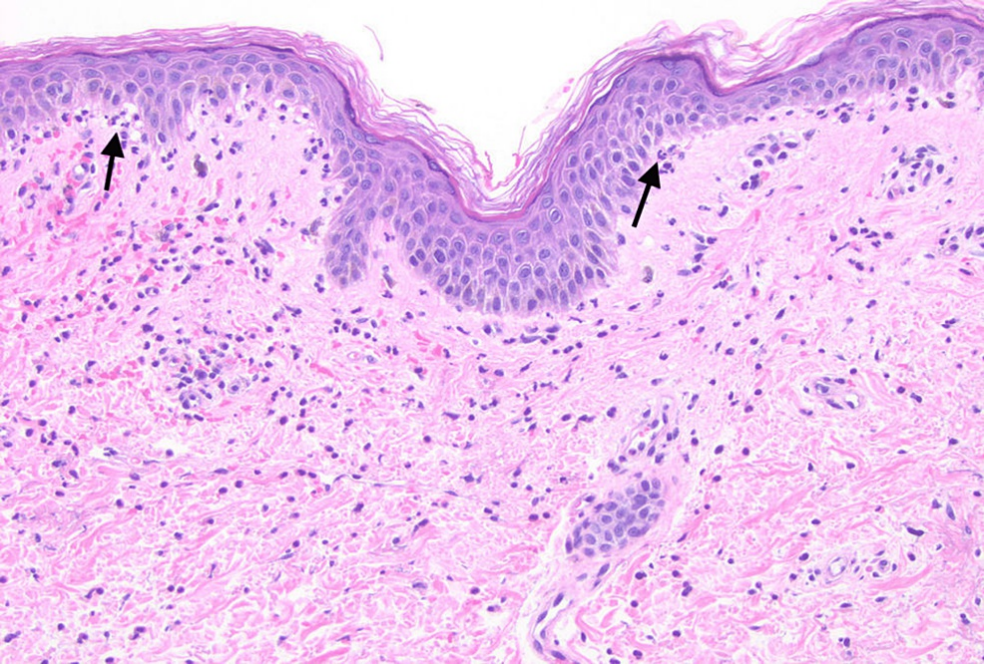
H&E stain of punch biopsy Observed are interface dermatitis with neutrophils and early subepidermal bullae formation. There is a vacuolar alteration with areas of separation associated with neutrophils noted in the dermoepidermal junction. H&E: Hematoxylin and eosin

Despite continued treatment, she later developed progressive lupus nephritis and vesiculobullous lesions on the forehead, dorsum of hands (Figure 1), arms, chest, neck, and back. A punch biopsy (Figure 2) of the lesions was consistent with BSLE. Direct immunofluorescence showed linear IgM, IgA, IgG, complement component 3 (C3), and albumin along the basement membrane. The patient was switched from azathioprine to mycophenolate 3000 mg. Despite increasing prednisone dosage again, there was no improvement in her skin lesions after a month. Glucose-6-phosphate dehydrogenase was within normal limits and dapsone 50 mg was started. After one week, the dapsone dosage was increased to 100 mg. Her skin lesions resolved in a few weeks without any residual scarring or pigmentation. As she developed hemolytic anemia after three months, dapsone was discontinued. The skin lesions did not recur.

## Discussion

Differentials for vesiculobullous lesions in lupus include bullous pemphigoid, dermatitis herpetiformis, and epidermolysis bullosa acquisita [6]. This is due to the similar histology and presentation. Epidermolysis bullosa acquisita has similar antibodies against collagen VII. It is more likely to have scarring and poor response to dapsone [7]. Dermatitis herpetiformis is histologically similar to BSLE except it has IgA deposition. Bullous pemphigoid can be differentiated with antibodies on the epidermal side [7]. The key to diagnosing this as BSLE in this patient is her history of lupus. 

Dapsone works well for mild BSLE with stable systemic lupus [6]. It usually resolves quickly with treatment. In this patient, dapsone was not used initially as the patient did not have stable lupus. Instead, the treatment recommendation is steroids for patients with active systemic lupus or moderate/severe BSLE [6]. Since the patient did not respond to previous management, dapsone was added. It is important to check for glucose-6-phosphate dehydrogenase deficiency before using dapsone as it is known to cause hemolytic anemia [8]. Dapsone dosage should be reduced to the lowest dosage with control of disease activity. Dapsone may not be required for over a year. Other options to treat BSLE include immunosuppressants like azathioprine, mycophenolate, or rituximab as second-line treatment [6]. The patient was previously on azathioprine and mycophenolate with no response to the skin lesions. These treatments are seen as second-line as they have not been proven to be as effective as dapsone for treating BSLE skin lesions [4]. Patients should be monitored for complications of BSLE including infection of the affected skin, esophagitis, multiorgan lupus flare, and medication side effects [7].

Bullous systemic lupus erythematous diagnosis criteria include the following: a previous diagnosis of lupus, vesiculobullous lesions, histopathology similar to dermatitis herpetiformis, negative or positive indirect immunofluorescence of basement membrane zone autoantibodies, and direct immunofluorescence showing IgG, IgM, or IgA at the basement membrane zone [8]. In our patient, 4/5 criteria were positive as this patient had a previous diagnosis of lupus, vesiculobullous lesions, histopathology similar to dermatitis herpetiformis, and direct immunofluorescence showing IgG, IgM, and IgA at the basement membrane zone. Autoantibodies to the basement membrane zone were not done due to availability. Sometimes a previous diagnosis of lupus might not be available as this can be the first presenting sign of lupus. As a result, it is important to rule out other vesiculobullous diseases.

Collagen VII usually anchors the epidermis to the dermis at the basement membrane zone. In BSLE, autoantibodies to collagen VII cause subepidermal blistering by weakening the basement membrane zone [8]. Laminin-5 and laminin-6 are anchoring filaments at the basement membrane zone. Autoantibodies to both laminin-5 and laminin-6 have been involved in BSLE [9]. This also plays a part in weakening the basement membrane zone causing subepidermal blistering. 

## Conclusions

Bullous systemic lupus erythematous is a very rare dermatologic manifestation of lupus. Rheumatologists should keep BSLE in mind as a diagnosis for patients with a history of lupus presenting with vesiculobullous lesions. In cases where the patient has stable lupus, dapsone should be the treatment of choice for treating these skin lesions. 
